# A Non-parametric Approach to the Overall Estimate of Cognitive Load Using NIRS Time Series

**DOI:** 10.3389/fnhum.2017.00015

**Published:** 2017-02-03

**Authors:** Soheil Keshmiri, Hidenobu Sumioka, Ryuji Yamazaki, Hiroshi Ishiguro

**Affiliations:** ^1^Hiroshi Ishiguro Laboratories, Advanced Telecommunications Research Institute InternationalKyoto, Japan; ^2^The Graduate School of Engineering Science, Osaka UniversityOsaka, Japan

**Keywords:** linear regression, curvilinear regression, working memory, near-infrared spectroscopy, mental workload prediction

## Abstract

We present a non-parametric approach to prediction of the n-back *n* ∈ {1, 2} task as a proxy measure of mental workload using Near Infrared Spectroscopy (NIRS) data. In particular, we focus on measuring the mental workload through hemodynamic responses in the brain induced by these tasks, thereby realizing the potential that they can offer for their detection in real world scenarios (e.g., difficulty of a conversation). Our approach takes advantage of intrinsic linearity that is inherent in the components of the NIRS time series to adopt a one-step regression strategy. We demonstrate the correctness of our approach through its mathematical analysis. Furthermore, we study the performance of our model in an inter-subject setting in contrast with state-of-the-art techniques in the literature to show a significant improvement on prediction of these tasks (82.50 and 86.40% for female and male participants, respectively). Moreover, our empirical analysis suggest a gender difference effect on the performance of the classifiers (with male data exhibiting a higher non-linearity) along with the left-lateralized activation in both genders with higher specificity in females.

## 1. Introduction

The advent of intelligent systems, capable of communicating with human (Yamazaki et al., 2007), introduces a tremendous opportunity to further explore some of most fundamental aspects of human society, thereby fathoming the intricacies exhibited in human behaviors pragmatically (Ogawa et al., 2011). Such systems have been increasingly proven to be of formidable potentials in investigation of foundational societal building blocks such as epigenetics (Prince and Gogate, 2007) and early child development (Lungarella et al., 2003; Tanaka et al., 2007). In this regard, communication is undoubtedly the foundation of sociability (Yamazaki et al., 2014). Research shows that a proper communication has direct and positive influence on physical (Sumioka et al., 2013) and mental (Yamazaki et al., 2016) health as well as quality of learning (Nakanishi et al., 2016).

Although it is crucial for these synthetic agents to be able to provide appropriate feedback on estimation of the brain activity of whose their operators are communicating with (Kumaran et al., 2016b), it is rather intractable to realize the internal state of cognitive activity of humans at highly sophisticated and complex behavioral level. Therefore, it is necessary to devise agents with mathematical models that are trained on basic cognitive activities, thereby providing them with adequate means to detect and/or measure such activities during interaction with human. Furthermore, it is of utmost important for these models to have the capacity for generalization and scalability on their available data, thereby reducing the time and effort that is, otherwise, required to interact with different individuals.

To this end, Near Infrared Spectroscopy (NIRS) presents an intriguing option for enabling these systems to act as timely and accurate analytical gateways into brain activity and emotional state of their human subjects. Cui et al. (2010b) define NIRS as a technology for functional brain imaging based on hemodynamic signals from the cortex. NIRS, in principle, is similar to functional magnetic resonance imaging (fMRI) (Cui et al., 2010a) without requiring the human subject laying motionless in the confined fMRI monitoring chamber. Its use for monitoring of brain activity becomes more attractive, considering the non-invasive operational setup of NIRS-related devices that are available at considerably lower cost along with their ease of use with portable, light-weighted headsets and their comparatively immunity to body movement (Dieler et al., 2012), unrestrictiveness, accessibility, as well as compact experimental setting (Moriai-Izawaa et al., 2012).

### 1.1. An overview of NIRS-based brain activity prediction

There exists a rich body of research pertaining to NIRS-based brain activity and emotional state classification. Naito et al. (2007) present communication means for patients struggling with amyotrophic lateral sclerosis (ALS) using quadratic discriminant analysis (QDA). Their model utilizes maximum amplitude and phase change as features to achieve an average accuracy of 80% on binary “yes/no” answers of forty male and female patients. Tai and Chau (2009) compares the performance of linear discriminant analysis (LDA) and support vector machine (SVM) on NIRS signals associated with the single-trail classification of the positively and negatively induced emotional tasks at individual level. Their results suggest that classification accuracy of these models vary with the length of the input signals. Luu and Chau (2009) apply linear discriminant analysis (LDA) on mean signal amplitude of NIRS data of nine human subjects to achieve an average accuracy of 80% on evaluating the choice of drinks among two available options in a single-trial scenario. Cui et al. (2010c) apply linear SVM on NIRS-related finger tapping task performed by six participants. Furthermore, they present an insightful investigation of the effect of the different feature spaces on classification accuracy. Their results suggest that features that provide the best classification for one dataset may not be optimal for all NIRS data, thereby suggesting their further optimization for individual participants. Holper and Wolf (2011) apply Fisher's linear discriminant analysis (FLDA) on motor imagery tasks of simple and sequential finger-tapping to report an average classification accuracy of 81.0% that is computed based on the classification performance of FLDA on NIRS data of the participants at the individual level. Hu et al. (2012) utilize contrast-to-noise ratio (CNR) as feature to decode deception on eight male subjects. They report classification accuracies of 83.44 and 81.14% using RBF and linear support vector machines (SVM), respectively. Furthermore, the accuracy of their model increases to 87.5% when applying their approach on an inter-subject setting (seven out of eight subjects). Naseer and Hong (2013a) use LDA on mean and slope of NIRS data as features to perform a left- and right-motor imagery by ten participants. Their approach achieves 73.35 and 83.0% accuracies on right- and left-wrist imagery tasks, respectively. Furthermore, they report an improvement in accuracy of their model by focusing on 2–7 s out of entire 10 s trials while extracting features, achieving average accuracies of 77.56 and 87.28% for right and left wrists, respectively. Herff et al. (2013a) apply LDA for binary discrimination between relax state and three different tasks (i.e., mental arithmetic, mental rotation, and word generation). They obtain 71% accuracy on mental arithmetic, 62% accuracy on mental rotation task, and 70% accuracy on word generation with respect to relax state on ten subjects. Nguyen et al. (2013) compare the performance of SVM in contrast with one-hidden-layer artificial neural network (ANN) for two hands tapping tasks performed by three human subjects. They use polynomial regression coefficients as features to report best average accuracy of 82.5% on right and left hands tapping of these subjects, using SVM. Furthermore, they obtain 85% on right and 75% on left hands tapping, using ANN. Herff et al. (2014) use fNIRS data along with LDA to classify between n-back (*n* ∈ {1, 2, 3}) and resting state to achieve up to 78% accuracy for single-trail discrimination. Naseer et al. (2014) compare the performance of LDA and SVM on online binary classification of mental yes/no answers (i.e., performing mental arithmetic vs. relax state in response to given questions) to report average classification accuracies of 74.28 and 82.14%, given the performance of these classifiers at the individual level. Xu et al. (2014) adopt χ^2^ statistic for feature extraction through discretization of NIRS data and apply linear SVM to achieve classification accuracy of 69–81% on right hand clench force motor imagery and clench speed motor imagery on six subjects. This article presents a useful literature review on the topic as well. Naseer and Hong (2015a) apply multi-class LDA for classification of the motor imagery based responses to four-choice questions (e.g., left-hand motor imagery to indicate option A) to report an accuracy of 73.3%, averaged on performance of their classifier at the individual level. Hong et al. (2015) use mean and slope of NIRS signal and multi-class LDA to classify between mental arithmetic, left hand motor imagery, and right hand motor imagery. They report an average accuracy of 75.6% on ten participants. Naseer et al. (2016) study the choice of optimal feature selection for binary classification of mental arithmetic and relax states, using LDA. Their results indicate that combination of the mean and the peak values of the signals associated with the individuals result in a significant improvement of the accuracy of their classifier. Naseer and Hong (2015b) present a comprehensive review of this topic.

### 1.2. Motivation and contributions

Despite impressive and promising results on classification of different brain activities using NIRS and fNIRS time series, all aforementioned approaches unanimously focus on improvement of the performance of different classification approaches at the individual (i.e., intra-subject) level, reporting their results that are averaged on the performance of these classifiers on single-participant basis. The major drawback of such an evaluation paradigm is the strong dependency of the accuracy of the adapted model on the performance of the individuals, thereby exhibiting high variation/bias. More specifically, there is a paucity of research on modeling and study of classification approaches that aim for generalization and scalability. Our approach addresses this issue via training on combined data of all participants (i.e., inter-subject level), thereby narrowing the gap between intra- and inter-subject brain activity prediction. It is apparent that such an approach facilitates the deployment and integration of these models in real-time systems since their learning mechanism is independent of the individual that they interact with.

Kamran and Hong (2014) argue that the NIRS time series data is a linear combination of various components, ranging from dynamical characteristics of the oxy-(HbO) and deoxy-hemoglobin (HbR) changes in a specific brain region and the influence from previous stimuli, to the physiological signals that prevail such time series data, and the baseline effect. This claim is further supported by Cui et al. (2010c) whose comparative analysis suggest that the slope (i.e., a linear correlate) of the NIRS data forms an important and highly informative feature in comparison to various feature spaces. These results explain the emergence of linear classifiers as dominant approaches to brain activity detection based on NIRS time series as presented in Section 1.1.

We take this observations and results into consideration while formulating our novel approach to brain activity prediction. In cognitive psychology, cognitive load refers to the total amount of mental effort utilized by the working memory while conducting a mental activity (Sweller, 1988). As such, the mental workload classification refers to the ability to distinguish between various level of brain activity that are pertinent to the same family of working memory through mathematical modeling of their corresponding time series data. In particular, we address the prediction of n-back task (Kirchner, 1958) as a proxy measure of mental workload. The n-back task is a continuous performance assessment, frequently used in cognitive neuroscience, to measure the working memory capacity (Gazzaniga et al., 2014). In this setting, the human participant is presented with a sequence of stimuli and the task consists of indicating when the current stimulus matches the one from n steps earlier in the sequence. The simple operational principles of such tasks provide opportunity to model changes in mental workload of human subjects through analysis of the effect of their level of difficulty on NIRS-related patterns of brain activity. Our study and its subsequent results focus on training a model on labeled data of human participants performing 1- and 2-back tasks, thereby distinguishing between these tasks during their prediction to infer the level of task difficulty based on its effect on mental workload. Our contributions are as follows:

1. We introduce a novel non-parametric approach to NIRS-based brain activity prediction that specifically exploits the intrinsic linearity exhibited by NIRS time series. Moreover, we demonstrate its correctness and convergence through analysis of its mathematical formulation. Our empirical results suggest that our model significantly improves upon prediction accuracy of n-back task as a proxy measure of mental workload using NIRS time series.
2. We introduce the potential that the utilization of differential entropy (DE) as a feature can offer to the solution concept of NIRS-based mental workload classification. Our experimental results suggest that DE empower a certain class of classifiers to achieve a higher prediction accuracy, compared to other commonly employed feature spaces. To the best of our knowledge, this is the first time that the utilization of DE in contrast with other NIRS-related feature spaces is reported in the literature. Moreover, these results are based on combined data of all participants (i.e., inter-subject level) and therefore the learned model is independent of data associated with any individual included in our experiment.
3. We provide empirical evidence on effect of the gender differences on mental workload prediction accuracy during n-back task through a comprehensive analysis of the results obtained by our model as well as a broad range of classifiers that are dominantly applied to NIRS-based prediction problem. This observation is in accord with the results in the literature on gender-specific brain activities (Weiss et al., 2003; Haut and Barch, 2006; Li et al., 2010).

The remainder of this article is organized as follows. We elaborate on formulation of our approach in Section 2. Section 3 provides details on data acquisition and experimental setup along with the data preprocessing and feature extraction steps. Results and comparative study of our model in contrast with state-of-the-art techniques in NIRS literature are presented in Section 4. Section 6 presents conclusion and some insight on the future direction of this research.

## 2. Methdology

Without loss of generality, let 𝕋_1_ and 𝕋_2_ represent two task spaces with the labels of their members being zero and one, respectively. Moreover, let ${\vec{\text{p}}}^{\left({𝕋}_{j}\right)},\;j=1,2$ be a feature vector in *jth* task space. Given the labels associated with these task spaces, we calculate their expected ratio of dissimilarity as:

(1) $$\begin{array}{l} r=\frac{E[\|{\vec{\text{p}}}_{i}^{\left({𝕋}_{1}\right)}\|]}{E[\|{\vec{\text{p}}}_{j}^{\left({𝕋}_{2}\right)}\|]},\;\forall {\vec{\text{p}}}_{i}\in {𝕋}_{1},\;\forall {\vec{\text{p}}}_{j}\in {𝕋}_{2} \end{array}$$

where *E*[.] returns the expected value of its argument (in this case, the mean of the array of Euclidean distances of feature vectors of respective task spaces) and ||.|| gives the norm of its input vector i.e., the norm of feature vector ${\vec{\text{p}}}_{i},\;i=1,\ldots N\in {\mathbb{R}}^{n},\;\left(n\geq 1\right)$ of the *jth* task space, with *N* representing the task space cardinality. We use this ratio to broaden the dissimilarity between 𝕋_1_ and 𝕋_2_:

(2) $$\begin{array}{l} {\vec{\text{p}}}_{i}=\{\begin{array}{ll} r\times {\vec{\text{p}}}_{i}\; & {\vec{\text{p}}}_{i}\in {𝕋}_{1}\\ max(\tau ,1.0-r)\times {\vec{\text{p}}}_{i},\; & \;otherwise \end{array} \end{array}$$

with τ being a threshold to reduce the squashing effect of second term in *max* function. It is worth noting that the effect of such a scaling of the original distribution of the elements of task spaces resembles approaches that seek for discriminative subspaces where the variance for one class is maximized while minimizing the variation in the second class (Fukunaga and Koontz, 1970; Kang and Choi, 2012). However, it differs from these approaches in that it captures a crude dissimilarity representation of these task spaces, thereby avoiding rather more computationally involved steps to define such dissimilarity. Although, *r* acts as an scaling factor between the two task spaces to refine their boundary via manipulation of their relative spatial distributions with respect to one another given their intrinsic dissimilarities and without modification of their inherent distribution (please refer to the Remark below), we use τ = 0.5 in present implementation to limit the effect of *r* if *E*[𝕋_2_] ≫ *E*[𝕋_1_].

Remark 1. Application of the expected ratio of dissimilarity *r* on between-task individual data elements preserves the originality of data. This is evident through the observations that:

1. *r* = 0: This scenario implies that at least one of the task spaces is an empty set, thereby indicating that no distinction is necessary.
2. *r* = 1: This occurs if and only if 𝕋_1_ and 𝕋_2_ represent the same data, a contradiction to existence of two task spaces.
3. *r* ∈ ℝ & *r* ≠ 0 & *r* ≠ 1: Equation (2) implies an affine transformation on all members of the same task space to uniformly scale these members as $f=\left\{{𝕋}_{i}\to {{𝕋}^{\prime }}_{i}\;|\;\vec{p}=\alpha \times \vec{p}+\beta \right\},\;\forall \vec{p}\in {𝕋}_{i},\;i=1,2$ with β = 0 and α = *r* or α = *max*(τ, 1.0 − *r*), given the task space. Moreover, *r* has an intrinsic property to scale the different task spaces in opposing directions as it is evident in Equation (2). Additionally, such an scaling factor follows the same direction for members of the same class, preserving their overall within-class distribution.

After scaling the data of the task spaces through the application of their dissimilarity ratio in Equations (1) and (2), we compute the respective geometric median (Lin, 1992; Fletcher et al., 2009) of these task spaces with equally weighted data [i.e., $w_{i}=1,\;\forall {\vec{\text{p}}}_{i}\in {𝕋}_{j},\;j=1,2$ in Definition 7.1, Appendix 7 (Supplementary Material)]. The geometric median of a given task space is always closest to the maximally formed cluster of a given task space than its respective outliers, as shown in the following Proposition.

Proposition 2.1. *Given a Task space* 𝕋, *its calculated geometric median is closest to the cluster associated with its observations than its outliers [please refer to Appendix 5 (Supplementary Material) for the proof]*.

Lemma 2.2. *Given a Task space* 𝕋, *the cumulative sum of distances of $\forall {\vec{\text{p}}}_{i}\backslash \vec{\text{c}}\in 𝕋$ to the geometric median $\vec{\text{x}}\in 𝕋$ with respect to outliers $\forall \vec{\text{c}}\in 𝕋$ is minimum [please refer to Appendix 5 (Supplementary Material) for the proof]*.

### 2.1. Weight matrix computation and refinement of decision boundary

Let *X* represent the input feature matrix that corresponds to the combined data of task spaces 𝕋_1_ and 𝕋_2_. Furthermore, let *y* be the row vector associated with *X* whose *ith* row entry represent the label of the *ith* feature vector in *X*. The weight vector that maps *X* onto *y* using the normal equation is (Cormen et al., 2001):

(3) $$\begin{array}{l} W={\left(X^{𝕋}X\right)}^{-1}X^{𝕋}y \end{array}$$

Let ${\vec{\text{x}}}_{1}\in {𝕋}_{1}$ and ${\vec{\text{x}}}_{2}\in {𝕋}_{2}$ be the geometric medians of task spaces 𝕋_1_ and 𝕋_2_, respectively. We compute the midpoint of these task spaces as a mean of their corresponding geometric medians with respect to their coordinates (i.e., their respective feature vectors):

(4) $$\begin{array}{l} \vec{\text{x}}=\frac{1}{2}(\chi_{{\vec{\text{x}}}_{1}}^{\left(j\right)}+\chi_{{\vec{\text{x}}}_{2}}^{\left(j\right)}),\;j=1,\ldots ,|{\vec{\text{x}}}_{i}|,\;i=1,2 \end{array}$$

with $\chi_{{\vec{\text{x}}}_{i}}^{\left(j\right)}$ being the *jth* coordinate (i.e., feature) of geometric median of the *ith* task space, i.e., ${\vec{\text{x}}}_{i}\in {𝕋}_{i},\;i=1,2$ and |.| returns the cardinality of its argument. Given $\vec{\text{x}}$ and the weights *W* in Equation (3), the new Sigmoidal boundary condition for 𝕋_*i*_, *i* = 1, 2 is:

(5) $$\begin{array}{l} \beta =\frac{1}{1+e^{-(W^{𝕋}\vec{\text{x}})}} \end{array}$$

i.e., new boundary condition, β, is obtained through application of Sigmoid activation function on inner product of weight vector *W* and midpoint $\vec{\text{x}}$. We utilize β to predict the labels of new data as:

(6) $$\begin{array}{l} y_{i}=\{\begin{array}{l} 1\;\frac{1}{1+e^{-(W^{𝕋}\vec{\text{p}})}}\geq \beta \\ 0\;otherwise \end{array} \end{array}$$

with $\vec{\text{p}}$ being the new feature vector associated with the recently generated input NIRS data.

Claim 2.3. *The midpoint of the geometric medians of the two task spaces* 𝕋_1_ *and* 𝕋_2_, *defines the most linearly optimal boundary between them [please refer to Appendix 5 (Supplementary Material) for the proof]*.

## 3. Preliminaries

### 3.1. Data acquisition and experimental setup

**Figure 1:**
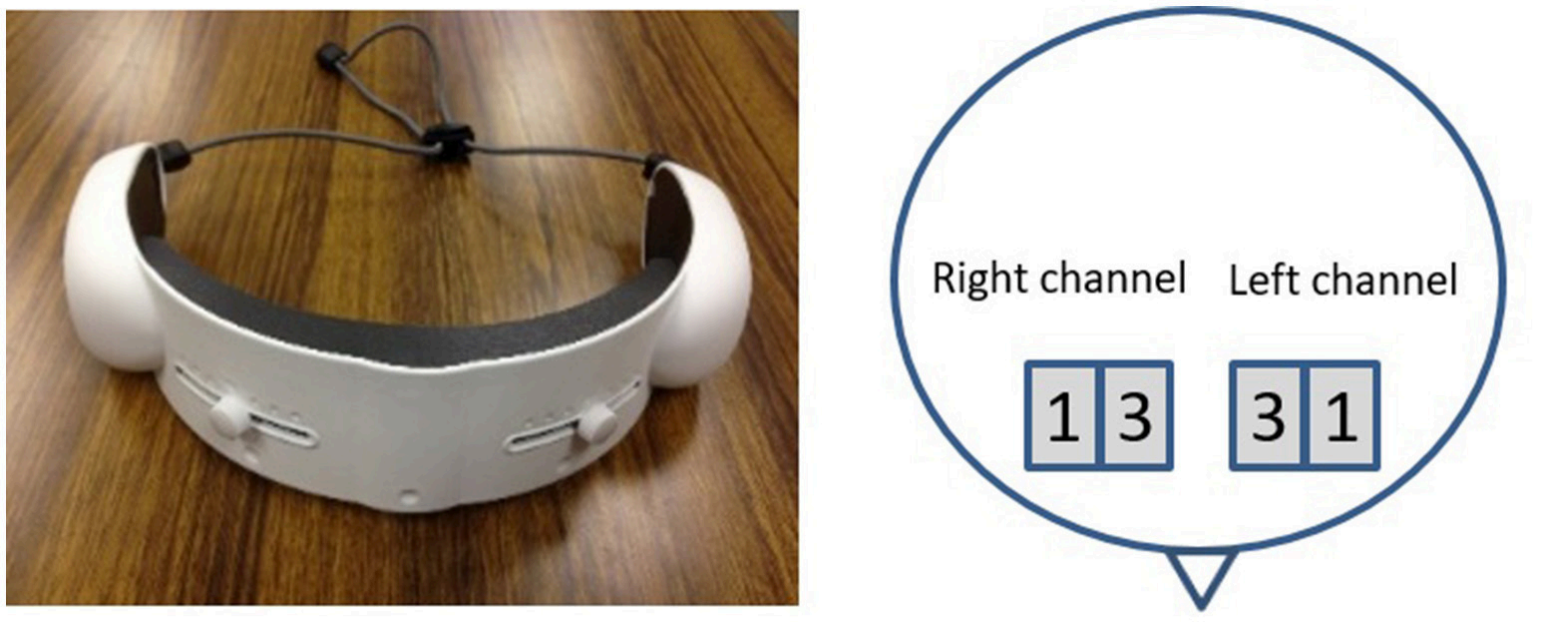
**The NIRS device used during the N-Back task (left)** along with the schematic of the locations of the left and right channels associated with the data collection procedure during the experiments **(right)**. The numbered squares refer to the left and right channels of depth 1.0 and 3.0 cm that are considered in this study, respectively.

Twenty-eight healthy right-handed volunteers (11 male and 17 female, *M* = 30.96 years, *SD* = 10.84) participated in the experiment. Prior to data collection, we received approval from the ethical committee at Advanced Telecommunications Research Institute International (Approval Code: 16-601-1), along with the informed consent from all participants. The data is acquired with a wearable optical topography system “HOT-1000,” developed by Hitachi High-Technologies Corporation (please refer to Figure 1). It is wore on the forehead of participants and collects data through four channels (i.e., *Left*_1_, *Left*_3_, *Right*_1_, and *Right*_3_, as shown in Figure 1). Furthermore, it allows for recording of the measurement of brain activity through detection of total blood flow via emitting a wavelength laser light (810 nm) at the 10 Hz sampling rate. The participants were requested to sit in front of a screen where the on-screen 1- and 2-back instructions (please refer to Section 1.2 for details) are presented. We use the FLANDERS (Nicholls et al., 2013) handedness questionnaire to measure the skilled hand preference of the participants. After a resting period of 1 min, they were instructed to focus on the voice, listening to a sequence of numbers in two separate tasks, clicking on the left mouse bottom if they recognize a repeated number meeting the 1- or 2-back repetition in the first and the second tasks, respectively.

### 3.2. Data preprocessing

First, we normalize the data corresponding to the four NIRS channels via subtracting the mean of the 1 min resting period as a baseline from this data. Next, we apply a 5-degree polynomial butter worth filter on each channel with 0.01 and 0.6 Hz for low and high bandpass, respectively. This is followed by linear detrending of the time series signals associated with each of these four channels. Lastly, we apply a 2-degree polynomial non-linear detrending.

**Figure 2:**
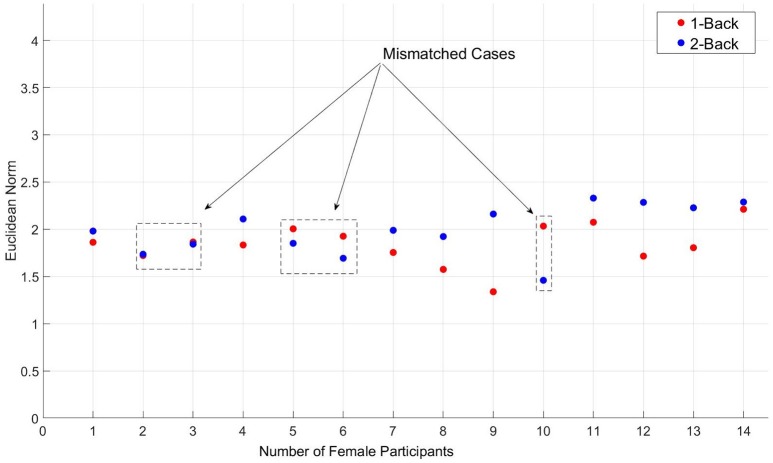
**Segmented (depth ***d*** = 1, as described in proofs 3.1 and 3.1.1) representation of the Euclidean norm distribution of NIRS data, corresponding to 1-back (red-colored circles) and 2-back (circles in blue) tasks - Female participants only (seven out of seventeen randomly selected)**. Cases that do not follow the general trend are indicated by dashed-line rectangles in this figure.

It is customary in NIRS data preprocessing to apply segmentation on the original data of participants, thereby increasing the size of samples that are, in most cases, small. However, we strongly believe that such segmentations degrade the performance of any supervised classifier, preventing its true accuracy to be estimated. Figure 2 shows the Euclidean norm distribution of NIRS data associated with 1-back (red-colored circles) and 2-back (circles in blue) tasks of seven randomly selected female participants in our study. In this figure, there are a number of participants whose data do not follow the general trend, namely, having their 2-back Euclidean norms above those associated with 1-back task. Although such misleading data are customary in many applications, segmentation of such cases introduces a rather redundant source of misclassification by prediction models. In fact, the negative effect of segmentation on estimation of true accuracy of any supervised classifier is significant as shown below.

Theorem 3.1. *Segmentation reduces the accuracy of any supervised classifier by a factor that is exponential to the depth of segmentation [please refer to Appendix 5 (Supplementary Material) for the proof]*.

Corollary 3.1.1. *Segmentation reduces the accuracy of any supervised classifier by $\frac{1}{2}\times s^{\left(d-1\right)}$ in worst case scenario [please refer to Appendix 5 (Supplementary Material) for the proof].*

### 3.3. Adopted feature spaces

Features can be directly extracted from raw NIRS data (Power et al., 2010). Alternatively, they are extracted from data after its transformation into hemoglobin concentration using Beer-Lamberts law (Hong et al., 2015). Luu and Chau (2009) show that effect of these two feature extraction strategies on prediction accuracy is insignificant. Moreover, Power et al. (2010) argues that the use of raw data for extracting features facilitates the integration of models into real world setting due to its less computational intensity. We adapt the same perspective for feature extraction in this article.

We compute separate sets of identical features for each of the four channels of our NIRS data. More specifically, we extract mean and slope of the signal (i.e., SM and SS, respectively) (Hong et al., 2015), contrast-to-noise-ratio (CNR) (Hu et al., 2012), and the moving average (Luu and Chau, 2009). In addition, we calculate the differential entropy (DE) of the data associated with these channels [please refer to Appnedix 6 (Supplementary Material)]. Although, DE is used as a feature in classification of brain activity and emotional state estimation based on electroencephalogram (EEG) data (Herff et al., 2013b; Shi et al., 2013; Kumaran et al., 2016a), this is the first time, to the best of our knowledge, that it is utilized for NIRS-based brain activity prediction. While calculating features, we divide the stream of NIRS data that correspond to each channel into four equal length sub-streams. Next, we compute the respective features for each of these sub-streams. This result in a four-dimensional feature vector in case of CNR, and DE for every channel. It is apparent that it is an eight-dimensional vector in case of SM and SS.

## 4. Case studies

### 4.1. Comparison strategy

We compare the performance of our approach in contrast with the prominent state-of-the-art techniques in NIRS-based classification. An overview of the literature pertinent to NIRS-based classification reveals that linear discriminant analysis (LDA) (Herff et al., 2013a; Naseer and Hong, 2013b; Hong et al., 2015), linear support vector machine (SVM) (Cui et al., 2010c; Hu et al., 2012; Hai et al., 2013), and quadratic discriminant analysis (QDA) (Naito et al., 2007) are dominant approaches that are adopted by the research community in this field. This is mainly due to the underlying linear trends of various components (e.g., oxy-(HbO) and deoxy-hemoglobin (HbR) changes in a specific brain region, etc.) that form the NIRS data time series (Cui et al., 2010c; Kamran and Hong, 2014). However, in addition to these methodologies, we include the comparative analysis of our approach in contrast with logistic regression (Freedman, 2009), RBF SVM (Chang et al., 2010), k-nearest-neighbor (KNN) (Fix and Hodges, 1951), decision tree (Breiman et al., 1984), random forest (Shi et al., 1995), and Naive Bayes (Stuart and Norvig, 2003) algorithms to ensure a thorough analysis of the performance of our model. We use Python scikit-learn^1^ package for this purpose. It is worth noting that the best setting of the parameters of these models are *K* = 3, *d* = 3, *n* = 10, *c* = 1e5 for number of neighbors in KNN, depth in decision tree, number of estimators in random forest, and penalty term in logistic regression, respectively. Furthermore, the penalty terms for linear and RBF SVM are *c* = 0.025 and *c* = 1.0, respectively.

### 4.2. Results collection

Algorithm 1 summarizes the procedure for acquiring the average prediction accuracy of a given classifier during the experiment. More specifically, we adopt a percentage-wise, N-Fold cross-validation strategy, starting with assigning 90% of total number of participants in random and without replacement (indicated by *split* variable) for training and finishing with splitting the data into half between train and test sets in a 5% countdown steps (line 14) which results in nine times of splitting in total. While assigning subjects for training and testing, we ensure that all data corresponding to a given participant is entirely assigned to one of these two sets, thereby preventing any potential similarity and/or shared representation of the individual information affecting/biasing the prediction accuracy of the classifiers. For each of these splits, we perform the prediction by a given classifier, ℂ, and collect its estimate, for a total number of 20 rounds (i.e., lines 6 through 9). We follow these procedure for every calculated feature (please refer to Section 3.3) and on every four NIRS channels. Finally, we report the best average prediction accuracy of the classifier, along with the type of feature and the channel leading to this result.

**Algorithm 1:**
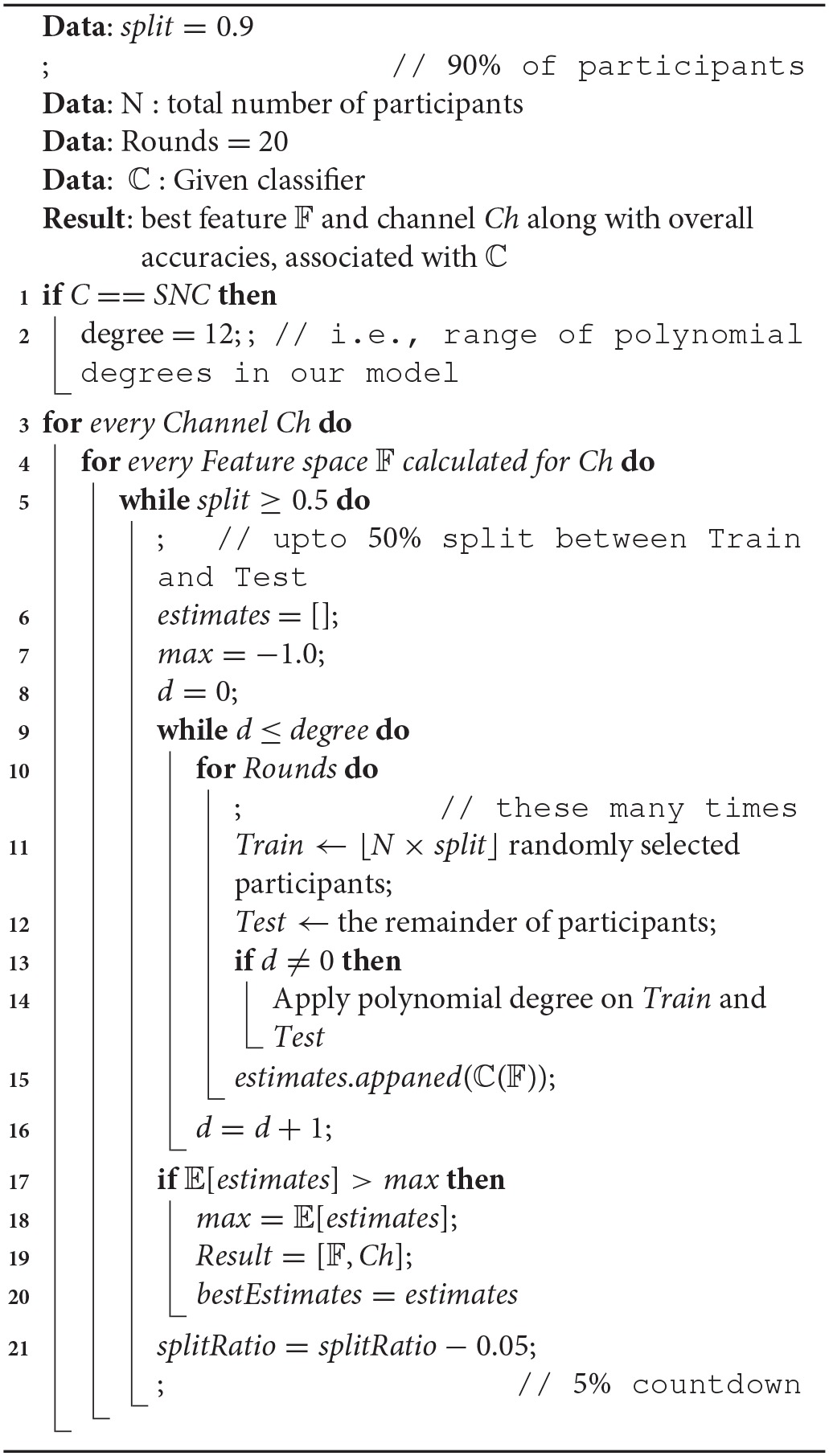
Percentage-wise, N-Fold Cross-Validation

It is worth noting that we include an additional step in case of our model to compute the best number of polynomial features to our model (i.e., line 9 in this algorithm). More specifically, we add a brute-force step in Algorithm 1 to add a polynomial feature to the input feature matrix *X* in Equation (3). The degree of this polynomial features is selected from the range [0, 12] with 0, indicating the original feature matrix *X* and without addition of any polynomial feature. We can afford this extra polynomial degree evaluation on our model due to its overall low-cost computational complexity, as outlined in Appendix 8 (Supplementary Material). Considering this procedure, there are 4(*channels*)×4(*features*)×9(*random splits*)×20(*repetition of random splits*) = 2880 steps involved to obtain the accuracy of each of the comparative classifiers. These steps increase to 2880 × 12(*polynomial feature selection*) = 34, 560 in case of our approach [indicated as SNC i.e., Sigmoid-Normal form Classifier due to the normal form regression in Equation (3)].

### 4.3. Performance results

This section provides details on performance results of our proposed approach in comparison with the selected classification strategies, outlined in Section 4.1. We present the average prediction accuracy of these techniques that are acquired through the steps described in Algorithm 1, along with the precision, the recall, and the F1-score of these classifiers. In addition, we outline the channel type and the type of feature, leading to their best average performances, respectively. Furthermore, we apply statistical analysis on these results to realize the degree of statistical significance in their performance differences. While conducting these comparative analyses, we consider three different settings of data, thereby empirically investigating the effect of gender difference on verbal working memory task (Li et al., 2010). More specifically, we consider the data associated with female only, data associated with male only, and the combined data of male and female participants.

#### 4.3.1. Female participants

Table 1 shows the average performance accuracy of different classifiers on the NIRS data pertinent to the female participants in our 1- and 2-back workload prediction. It is worth noting that we assign the positive label to 2-back tasks during the predictions. Furthermore, we use the “precision_score,” the “recall_score,” and the “f1_score” from scikit-learn to compute the precision, recall, and F1-score of these classifiers. Entries “Feature” and “Channel” refer to the NIRS data channel and type of the feature that are preferred by each model, respectively. Furthermore, “Deg.” shows the number of polynomial degree features that are selected by our model. This entry is hyphenated for other classifiers as it is not applied to their settings. In addition, we abbreviate our approach as SNC which stands for Sigmoid-Normal form Classifier where the term Normal form refers to the normal form regression in Equation (3) (Cormen et al., 2001).

**Table 1:** **Female participants—average performance accuracy of our model in contrast with K-neatest-neighbor (KNN), Linear SVM, RBF SVM, Decision Tree, Random Forest, Naive Bayes, Linear Discriminant Analysis (LDA), Quadratic Discriminant Analysis (QDA), Logistic Regression (Logistic reg)**.

**Classifier**	**Accuracy (%)**	**Precision**	**Recall**	**F1-score**	**Feature**	**Channel**	**Deg**.
SNC	82.5	0.85	0.90	0.84	DE	*Left*_1_	0
KNN	77.5	0.81	0.75	0.76	DE	*Left*_1_	–
Linear SVM	65.0	0.61	0.66	0.60	Moving Avg	*Left*_1_	–
RBF SVM	75.0	0.73	0.87	0.76	DE	*Right*_1_	–
Decision tree	77.0	0.81	0.75	0.75	DE	*Left*_1_	–
Random forest	74.29	0.83	0.59	0.67	DE	*Left*_1_	–
Naive bayes	80.0	0.78	0.90	0.80	Moving Avg	*Left*_1_	–
LDA	78.0	0.88	0.70	0.76	DE	*Left*_1_	–
QDA	80.0	0.87	0.78	0.80	DE	*Left*_1_	–
Logistic reg	77.5	0.8	0.86	0.80	DE	*Left*_1_	–

*SNC entry represent the results obtained by our model. DE and Moving Avg are the differential entropy and the moving average features*.

**Figure 3:**
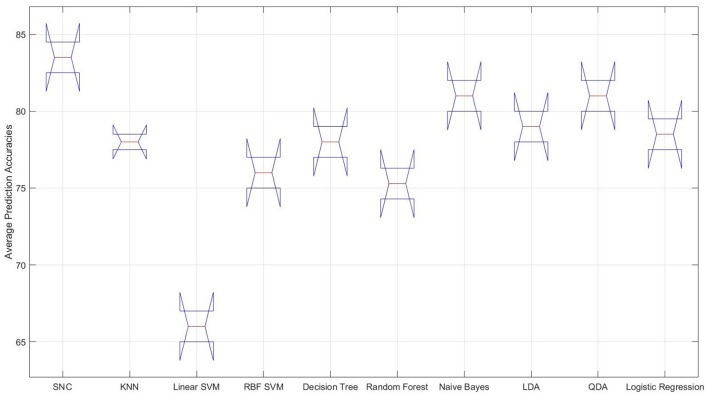
**Female data—distribution of the overall averaged prediction accuracies of the classifiers**. From left to right: our approach (SNC), KNN, linear SVM, RBF SVM, naive Bayes, LDA, QDA, and logistic regression. It is apparent that the performance of the linear SVM is significantly poorer than other classifiers on NIRS data associated with the female participants.

It is interesting to observe that differential entropy (i.e., DE entries in Feature column of this table) is the feature that is predominantly selected by the classifiers. The only exceptions are the linear SVM and naive Bayes classifiers that both choose the moving average as their preferred choices of feature. However, the overall poor performance of linear SVM as shown in Figure 3 suggests that it is not a good choice for prediction of such mental tasks. As a result, its choice of feature as an indicative of strength of moving average is not warranted. Moreover, *Left*_1_ is the channel of choice for majority of the classifiers. The only exception to this observation is the RBF SVM. Furthermore, the “Average Accuracy” entry of Table 1 indicate that, given the procedure elaborated in Algorithm 1, the performance of our model on average, using the NIRS data of female participants outperforms all the other classifiers. More specifically, the difference between these averages is above one standard deviation (*SD* = 4.76). Moreover, this observation is supported by the multiple comparison ANOVA using Bonferroni on the average accuracies of all steps involved in Algorithm 1 (*p* < 0.00002, *F* = 24.44, *SD* = 1.41)^2^. Figure 3 shows the distribution of these average prediction accuracies that are exhibited by each model. It is apparent in this figure that, all the classifiers achieve an above 75% accuracy on their overall averaged predictions. The only exception is the linear SVM that performs significantly below this trend. Moreover, this figure shows that naive Bayes and QDA are the closest to our model (*p* < 0.00011, *t* = 97.0, *SD* = 1.43, one-sample *t*-test).

#### 4.3.2. Male participants

Table 2 corresponds to the average performance accuracy of the classifiers on NIRS data pertinent to the male participants. “SM and SS” refers to the signal mean and signal slope features (please see Section 3.3). In this table, the first observation to note is the non-uniformity on preferred type of feature by different models. However, two out of three classifiers with highest average prediction accuracies i.e., our approach (SNC) and logistic regression prefer DE (the third one is naive Bayes that chooses moving average as its preferred feature space). The same observation hold valid in case of the channel selection where the number of models with *Left*_1_ as their preferred choice is comparably smaller than those in female case. However, it is still the dominant trend (five out of ten with *Left*_3_ and *Right*_3_ being selected one and four times, respectively). Furthermore, our model prefers an increase in its polynomial features, adopting a four degree polynomial for its input features, compared to female case in Table 1.

**Table 2:** **Male participants—average performance accuracy of our model in contrast with K-neatest-neighbor (KNN), Linear SVM, RBF SVM, Decision Tree, Random Forest, Naive Bayes, Linear Discriminant Analysis (LDA), Quadratic Discriminant Analysis (QDA), Logistic Regression (Logistic reg)**.

**Classifier**	**Accuracy (%)**	**Precision**	**Recall**	**F1-score**	**Feature**	**Channel**	**Deg**.
SNC	86.4	0.87	0.94	0.87	DE	*Left*_1_	4
KNN	73.3	0.65	0.78	0.70	SM and SS	*Left*_3_	–
Linear SVM	70.0	0.65	0.75	0.67	Moving Avg	*Left*_1_	–
RBF SVM	76.0	0.80	0.74	0.75	Moving Avg	*Left*_1_	–
Decision tree	77.5	0.84	0.67	0.71	SM and SS	*Right*_3_	–
Random forest	77.50	0.83	0.67	0.71	SM and SS	*Right*_3_	–
Naive bayes	80.0	0.78	0.90	0.80	Moving Avg	*Left*_1_	–
LDA	78.33	0.81	0.81	0.79	Moving Avg	*Left*_1_	–
QDA	75.0	0.78	0.72	0.71	DE	*Right*_3_	–
Logistic reg	80.0	0.78	0.75	0.74	DE	*Right*_3_	–

*SNC entry represent the results obtained by our model. DE, SM, and SS, and Moving Avg are the differential entropy, the signal mean and slope, and the moving average features*.

**Figure 4:**
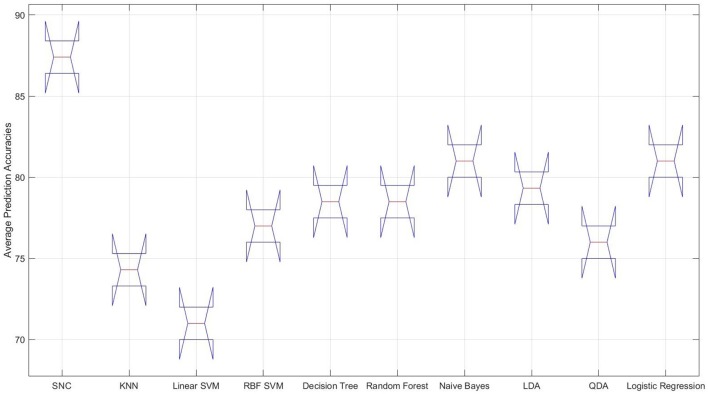
**Male data—distribution of the overall averaged prediction accuracies of the classifiers**. From left to right: our approach (SNC), KNN, linear SVM, RBF SVM, naive Bayes, LDA, QDA, and logistic regression. It is apparent that the performance of the linear SVM considerably poorer than other classifiers on NIRS data associated with the female participants.

Our model achieves a significantly higher result compared to other classifiers, as it is evident in Table 2 and Figure 4. Additionally, it improves upon its performance on female data significantly (*p* < 0.014, *t* = 43.31, *SD* = 2.76, one-sample *t*-test). Furthermore, it obtains higher precisions and recalls, resulting in better F1-score than its average performance on female data, as the comparison of these entries in Tables 1, 2 suggests. Moreover, Figure 4 shows the significant improvement on overall averaged prediction accuracy that is achieved by our model in comparison with other classifiers which is supported by multiple comparison ANOVA with Bonferroni (*p* < 0.00004, *F* = 19.41, *SD* = 1.41).

#### 4.3.3. Combined data of female and male participants

Table 3 presents the results obtained by these algorithms on combined data of male and female participants. Although our model is significantly improving upon prediction accuracies in comparison with other classifiers (*p* < 0.000009, *F* = 26.14, *SD* = 5.11, one-way ANOVA with “bonferroni”), it is apparent that combined data of different genders has a negative effect on average performance of all these algorithms. More specifically, the average accuracy of these algorithms is significantly worsened once the data of male and female participants are combined (MEAN = 13.0, *SD* = 4.77 and MEAN = 13.79, *SD* = 3.29 with respect to female only and male only data). Our proposed model shows an 11.07% decay in its average accuracy. This is followed by a significant increase in its choice of polynomial degree, from 0 and 4 in female and male only cases, respectively, to 9 in case of combined data. It is worth noting that such an increase in preferred polynomial feature degree (MEAN = 4.33, *SD* = 4.51) indicates a significant increase in non-linearity exhibited by the combined data of different genders. However, it continues with *Left*_1_ and *DE* as its best choice of channel and the selected feature as in previous data settings. The degradation of the average accuracy is evident in second and third best performing classifiers in case of male only data (i.e., Naive Bayes 19.37% and logistic regression 13.33%) and female only (i.e., Naive Bayes 19.37% and LDA 13.00%).

**Table 3:** **Female and male participants—average performance accuracy of our model in contrast with K-neatest-neighbor (KNN), Linear SVM, RBF SVM, Decision Tree, Random Forest, Naive Bayes, Linear Discriminant Analysis (LDA), Quadratic Discriminant Analysis (QDA), Logistic Regression (Logistic reg)**.

**Classifier**	**Accuracy (%)**	**Precision**	**Recall**	**F1-score**	**Feature**	**Channel**	**Deg**.
SNC	75.33	0.75	0.81	0.76	DE	*Left*_1_	9
KNN	65.46	0.69	0.67	0.66	Moving Avg	*Left*_1_	–
Linear SVM	58.33	0.61	0.73	0.64	Moving Avg	*Left*_3_	–
RBF SVM	64.26	0.60	0.79	0.67	DE	*Left*_3_	–
Decision tree	63.75	0.74	0.47	0.53	SM and SS	*Right*_3_	–
Random forest	61.67	0.76	0.63	0.62	Moving Avg	*Left*_1_	–
Naive bayes	60.63	0.59	0.62	0.60	Moving Avg	*Left*_1_	–
LDA	65.00	0.67	0.71	0.67	CNR	*Left*_1_	–
QDA	57.08	0.57	0.52	0.53	DE	*Left*_3_	–
Logistic reg	66.67	0.68	0.80	0.72	Moving Avg	*Left*_1_	–

*SNC entry represent the results obtained by our model. DE, SM and SS, and Moving Avg are the differential entropy, the signal mean and slope, and the moving average features*.

Although the *Left*_1_ remains the dominant channel of choice while using the combined data in Table 3, *Moving Average* replaces *DE* as dominantly utilized feature by these models. We observe this shift in choice of feature from *DE* to *Moving Average* while comparing the *feature* entries in Tables 1, 2 as well. This suggests that the increase in non-linearity as well as change in adopted feature space in case of combined data is mainly due to the data associated with the male participants. Moreover, results of one-sample *t*-test on these accuracies indicate that such degradations on average accuracy of these models are significant (*p* < 0.00001, *T* = −8.66, *SD* = 4.76 with respect to female only and *p* < 0.0000007, *T* = −13.25, *SD* = 3.29 with regards to male only). This suggests that the gender difference introduces a significant impact on NIRS related brain activity while performing 1- and 2-back tasks.

## 5. Discussion

Table 1 indicates that our model (i.e., SNC), naive Bayes, and QDA achieve best accuracies on female participants, with SNC obtaining a significant improvement over the results of other two models. Moreover, the precision and recall entries of this table suggest that both SNC and naive Bayes have a better accuracy on predicting the 1-back as opposed to 2-back tasks. This is evident in their higher recall entries in this table, compared to their precision. However, this is reversed in case of QDA where it achieves a better prediction on 2-back task. In addition, result of one-sample *t*-test suggests that their performance differences on predicting these tasks are significant (*p* < 0.0012, *t* = 30.54 in case of precision and *p* < 0.0023, *t* = 21.50 for recall). Furthermore, the same trend is observed in case of male participants in Table 2, where SNC, naive Bayes, and logistic regression form the high performing classifiers, with SNC and naive Bayes having higher accuracy on 1-back tasks (i.e., higher recall) as opposed to logistic regression that obtains higher precision (*p* < 0.0015, *t* = 27.0 in case of precision and *p* < 0.0046, *t* = 14.92 for recall). This is a complementary result to Cui et al. (2010c), whose observation indicate that features that provide the best prediction for one data set may not be optimal for all NIRS datasets. More specifically, our result suggests that real time systems can benefit from ensemble models with classifiers that are primarily trained for and predominantly better in predicting a subclass of overall task spaces, resulting in significant improvement of performance on estimation of the brain activity of human subjects by the systems that they are deployed in. In addition, Tables 1, 2 suggest a gender difference effect on the performance of the classifiers, with male participants exhibiting a higher non-linearity in their NIRS data brain activity. This is evident in increase in number of polynomial features that are adopted by our model as we compare the “Deg.” entries of these tables. Moreover, we observe a decay in accuracies of all models on combined data of different genders in Table 3. These observations are in accordance with the analytical study of prefrontal cortex during a verbal working memory task (Li et al., 2010). In addition, the result of the literature on brain region activation during memory and language processing suggest a left-lateralized activation in both genders with higher specificity in females (Weiss et al., 2003; Haut and Barch, 2006; Li et al., 2010). Our empirical results is in accordance with the literature as indicated by predominant choice of *Left*_1_ NIRS channel by classifiers in Tables 1–3, with a higher preference on this channel while using female data.

## 6. Conclusion

We introduce a non-parametric approach to prediction of n-back task as a proxy measure of mental workload of human subjects using NIRS data. Our approach takes advantage of subtle underlying linearity exhibited by the components of the NIRS data to emphasize the idiosyncratic characteristics of brain activity through application of their dissimilarity. Furthermore, it adopts a one step regression strategy to compute its weights, thereby allowing our model to further explore the potential that is offered via introduction of polynomial features to further improve its accuracy.

We choose 1- and 2-back tasks as a typical proxy measure of mental workload to examine the prediction accuracy of our approach. The simple operational principles of such tasks provide opportunity to model changes in brain activity. The comparative analysis of the performance of our model in contrast with state-of-the-art techniques shows a significant improvement on prediction accuracy of these tasks. Furthermore, our results suggest that adaptation of differential entropy (DE) to compute features of NIRS data introduces a potential for extracting features that help increase the accuracy of certain class of learning algorithms. This is, to the best of our knowledge, the first time to utilize DE in NIRS-based prediction.

An interesting observation that is revealed through our results is the effect of gender differences on the performance of the classifiers. Whereas our approach achieves 86.40 and 82.50% on male and female participants, respectively, its accuracy reduces to 75.33% once data associated with different genders is combined. This suggests that devising real time systems with classifiers that take into account such gender specificity on the nature of signals corresponding to brain activity leads to higher accuracy of such systems while interacting with humans. Furthermore, such a degradation of the performance accuracy is exhibited by all the classifiers whose performance are studied in contrast with our proposed approach. Although our findings are supported by a number of analytical studies on the influence of gender on brain activation pattern and hemodynamics, this empirical observation is at its very early stage and drawing a definitive conclusion demands further statistical and experimental analyses.

In this study, we carry out our analysis on human subjects whose NIRS data are collected during real time sessions. However, results reported in this article are based on offline use of this data. Therefore, future of this research pertains to deployment of our model on real time system to determine its utility to the solution concept of state estimation of the brain activity of human subjects. Furthermore, it is crucial to increase the number of participants to acquire larger amount of data, thereby analyzing the effect of higher variation of brain activity patterns on the prediction accuracy of our model due to increase in amount of NIRS data.

We collect our results on the accuracy of our model in contrast with different classifiers while treating the NIRS channels independently. However, it is interesting to analyze the effect of the features that are calculated based on various combination of these channels on the overall accuracy of these classifiers in the future.

Another important factor that demands special consideration is to test the performance of our approach in scenarios with more than two classes of tasks (e.g., N-back task with *N* ≥ 3, up to an upper bound threshold), thereby evaluating its ability to generalize on more complex scenarios.

The prime target of our research is to provide synthetic agents with the ability to engage in meaningful communication with their human counterparts. We utilize n-back task as an intermediate, tractable approximation of underlying mental workload, necessary to conduct such highly complex communicational tasks. Therefore, we use the results acquired in this study as a basis to build a representational space based on which generalization on estimation of the brain activity of human subjects, in their broader perspectives, is foreseeable. Our future work will include deployment of our model in a real-world setting to realize the utility of our approach to the solution concept of human-robot interaction.

## Ethics statement

This study was carried out in accordance with the recommendations of the ethical committee of Advanced Telecommunications Research Institute International (ATR) with written informed consent from all subjects. All subjects gave written informed consent in accordance with the Declaration of Helsinki. The protocol was approved by the ATR ethical committee (approval code: 16-601-1).

## Author contributions

SK formulated the mathematical model, its correctness analysis, as well as conducting simulation and collecting results on performance of all the models involved for the purpose of comparative analysis in this article. Furthermore, he prepared the draft of the article. HS acted as research lead, designing the experiment, supervising the progress, and taking part in experimental setup while collecting data from participants. In addition, he reviewed the entire content of the article and provided insightful feedback to improve the quality of the writing as well as the results presented. RY designed the experiments and carried them out with the participants. Furthermore, he completed all the documentation associated with the experimental setup (e.g., collecting consents, research approval from ATR ethical committee, etc.) As the head of HIL, HI oversee the entire activity of all research teams and themes, ensuring the soundness of all proposals, quality of results, and their validity.

## Funding

This research is supported by Impulsing Paradigm Change through Disruptive Technologies Program (ImPACT): Actualize Energetic Life by Creating Brain Information Industries, Brain Robotics for communication, funded by the Japanese Cabinet Office.

### Conflict of interest statement

The authors declare that the research was conducted in the absence of any commercial or financial relationships that could be construed as a potential conflict of interest.

## Footnotes

^1^http://scikit-learn.org/stable/

^2^All statistical analyses reported in this article are based on MATLAB R2016a

## Proof

### A. Proposition 2.1

*PROOF*. Definition 7.1 in Supplementary Materials ensures that the cumulative sum of distances of $\forall {\vec{\text{p}}}_{i}\in 𝕋$ to its geometric median $\vec{\text{x}}\in 𝕋$ are minimized. In addition, it is the case that:

(A1) $$\begin{array}{l} \forall {\vec{\text{p}}}_{i}\setminus \vec{\text{c}}\in 𝕋:Q_{1}-1.5\times (Q_{3}-Q_{1})\;\leq \;\|{\vec{\text{p}}}_{i}\|\;\leq \;Q_{3}+1.5\times (Q_{3}-Q_{1}) \end{array}$$

and

(A2) $$\begin{array}{l} \forall \vec{\text{c}}\;\neq {\vec{\text{ p}}}_{i}\in 𝕋:\;\|\vec{\text{c}}\|\;<\;Q_{1}\;-\;1.5\;\times \;(Q_{3}\;-\;Q_{1})\;or\;\|\vec{\text{c}}\|\;>\;Q_{3}\\ \;+1.5\;\times \;(Q_{3}\;-\;Q_{1}) \end{array}$$

where $\vec{\text{c}},Q_{1},$ and *Q*_3_ are the outliers, the 25*th*, and the 75*th* quantiles associated with the distance distribution of $\forall \vec{\text{p}}\in 𝕋$, respectively. There are two cases to consider:

1. *Q*_1_, *Q*_3_ ∈ 𝕋: This implies that $Q_{j}={\vec{\text{p}}}_{i}\backslash \vec{\text{c}},\exists {\vec{\text{p}}}_{i}\in 𝕋$ or they cannot form the boundary condition for the outliers $\vec{\text{c}}\in 𝕋$. Therefore, *Q*_1_ and *Q*_3_ are the outer most data on the convex of $\forall {\vec{\text{p}}}_{i}\backslash \vec{\text{c}}\in 𝕋$. Moreover, Claims 7.1 and 7.2 in Supplementary Materials ensure that geometric median $\vec{\text{x}}\in 𝕋$ is within its convex, resulting in:
  (A3) $$\begin{array}{l} \|{\vec{\text{p}}}_{i}\;-\;\vec{\text{x}}\|\;<\;\|\vec{\text{c}}\;-\;\vec{\text{x}}\|,\;\forall {\vec{\text{p}}}_{i}\setminus \vec{\text{c}}\;\in \;𝕋,\;\forall \vec{\text{c}}\;\in \;𝕋 \end{array}$$
2. *Q*_1_, *Q*_3_ ∉ 𝕋: This implies that *Q*_1_ and *Q*_3_ are calculated using the lower and the upper tails of 𝕋 pertinent to its 25^*th*^ and 75^*th*^ quantiles. It is apparent that at most one of the two values involved in calculation of *Q*_1_ and *Q*_3_, respectively, is among outliers at the given percentile. Furthermore, these outliers (if existed) are the ones closer to two extreme tails of 𝕋. Using the non-outliers to form the convex of 𝕋, the remainder of the proof follows the previous case.

### B. Lemma 2.2

*PROOF*. There are two cases to consider:

1. Single Outlier: Let ${\vec{\text{p}}}_{1},\;\ldots ,\;{\vec{\text{p}}}_{N}\in 𝕋$ be the data that form the task space 𝕋. Without loss of generality, let $\vec{\text{c}},\;\vec{\text{x}}\in 𝕋$ represent the outlier and the geometric median associated with the task space 𝕋, respectively. Claims 7.1 and 7.2 in Supplementary Materials, imply that $\vec{\text{x}}$ is within the convex of data that corresponds to 𝕋. The pairwise cumulative sum of distances of ${\vec{\text{p}}}_{i}\backslash \vec{\text{c}}\in 𝕋$ to $\vec{\text{x}}\in 𝕋$ with respect to the outlier $\vec{\text{c}}\in 𝕋$ is:
  (A4) $$\begin{array}{l} \left(\|\vec{\text{c}}-\vec{\text{x}}\|\;+\;\|{\vec{\text{p}}}_{1}-\vec{\text{x}}\|)+\ldots +(\|\vec{\text{c}}-\vec{\text{x}}\|\;+\;\|{\vec{\text{p}}}_{N}-\vec{\text{x}}\|\right)\\ \;=\;\|\vec{\text{c}}-\vec{\text{x}}\|\;\times \;(\|{\vec{\text{p}}}_{1}-\vec{\text{x}}\|\;+\;\ldots \;\|{\vec{\text{p}}}_{N}-\vec{\text{x}}\|)\\ \;=\;\|\vec{\text{c}}-\vec{\text{x}}\|\text{​}\times \text{​}\sum_{i=1}^{N}\left\|{\vec{\text{p}}}_{i}-\vec{\text{x}}\right\|\;\geq \text{​​}\sum_{i=1}^{N}\left\|{\vec{\text{p}}}_{i}-\vec{\text{x}}\right\| \end{array}$$
2. Multiple Outliers: Let $C=\left\{{\vec{\text{c}}}_{1},\;\ldots ,\;{\vec{\text{c}}}_{m}\right\}$ be the set of outliers with m and N representing the number of outliers and total number of data associated with task space 𝕋, respectively. Following the case of single outlier, we have:
  (A5) $$\begin{array}{l} \;[(\|{\vec{\text{c}}}_{1}\;-\;\vec{\text{x}}\|\;+\;\|{\vec{\text{p}}}_{1}\;-\;\vec{\text{x}}\|)+\ldots +(\|{\vec{\text{c}}}_{m}\;-\;\vec{\text{x}}\|\;+\;\|{\vec{\text{p}}}_{1}\;-\;\vec{\text{x}}\|)]\\ +\ldots +[(\|{\vec{\text{c}}}_{1}\;-\;\vec{\text{x}}\|\;+\;\|{\vec{\text{p}}}_{N}\;-\;\vec{\text{x}}\|)\;+\;(\|{\vec{\text{c}}}_{m}\;-\;\vec{\text{x}}\|\;+\;\|{\vec{\text{p}}}_{N}\;-\;\vec{\text{x}}\|)]\\ \;=\sum_{i=1}^{m}\left\|{\vec{\text{c}}}_{i}\;-\;\vec{\text{x}}\right\|\;\times \sum_{j=1}^{N}\left(\right\|{\vec{\text{p}}}_{i}\;-\;\vec{\text{x}}\|\;\geq \;\sum_{j=1}^{N}\left(\right\|{\vec{\text{p}}}_{i}\;-\;\vec{\text{x}}\| \end{array}$$
  It is apparent that Theorem 3.1 and Corollary 3.1.1 hold as the cardinality of set *C* approaches *N*.

### C. Claim 2.3

*PROOF*. Let ${\vec{\text{x}}}_{1}\in {𝕋}_{1}$ and ${\vec{\text{x}}}_{2}\in {𝕋}_{2}$ be the two geometric medians. Claim 7.1 in Supplementary Materials, implies that they are within the convex of data associated with 𝕋_1_ and 𝕋_2_. Furthermore, Proposition 2.1 and Lemma 2.2 imply that each 𝕋_*i*_, *i* = 1, 2 has its data maximally clustered around its ${\vec{\text{x}}}_{i}$. Let $\vec{\text{x}}$ represent the midpoint of the line segment, connecting ${\vec{\text{x}}}_{1}$ and ${\vec{\text{x}}}_{2}$. Furthermore, let *L* be the line segment that passes through $\vec{\text{x}}$ and is orthogonal to $x\vec{{\;}_{1}}x_{2}$. This implies that $x\vec{{\;}_{1}}x_{2}$ and $x\vec{{\;}_{1}}x_{2}$ are the normals to *L* with respect to the task spaces 𝕋_1_ and 𝕋_2_, thereby maximally separating ${\vec{\text{x}}}_{1}$ and ${\vec{\text{x}}}_{2}$ from *L*.

### D. Theorem 3.1

*PROOF*. Let *s* represent the number of segments that each base stream is segmented to (e.g., *s* = 2 if the original stream is split into half). Furthermore, let *d* be the depth of segmentation (e.g., *d* = 2 if segmentation is applied on segmented data after the first step of segmentation). Given the original unsegmented data, it splits into *s* segments at depth *d* = 1, that are segmented into another *s* segments on their own at *d* = 2. Continuing in this fashion, we have *s*^*d*^ segments at depth *d*. Allowing for *m* to represent the number of mismatched cases in *s* segments at *d* = 1 i.e., the onset of segmentation, the degradation of the accuracy of a given classifier is $\frac{m}{s}\times s^{d}=m\times s^{\left(d-1\right)}$.

### E. Corollary 3.1.1

*PROOF*. If *m* ≪ *s* then $\frac{m}{s}\to 0$ as *s* → ∞, implying a negligible effect of such cases on the accuracy of a given classifier. On the other hand, $\frac{m}{s}\approx 1$ as *m* → *s*. Moreover, *m* ≠ *s* as it contradicts being mismatched cases in principle. Furthermore, it is apparent that at most $m=\frac{s}{2}$ (i.e., the maximum entropy) since any other case for proportionality between *m* and *s* is fixed by reversing their oder, thereby satisfying the *m* < *s*. Substituting for *m*, we get $\frac{\frac{s}{2}}{s}\times s^{\left(d-1\right)}=\frac{1}{2}\times s^{\left(d-1\right)}$

## References

[B1] Boltyanski V. Martini H. Soltan V. (1999). Geometric Methods and Optimization Problems. Boston, MA: Kluwer Academic.

[B2] Breiman L. Friedman J. Olshen R. Stone C. (1984). Classification and Regression Trees. Monterey, CA: Wadsworth & Brooks; Cole Advanced Books & Software.

[B3] Chang Y. Hsieh C. Chang K. Ringgaard M. Lin C. (2010). Training and testing low-degree polynomial data mappings via linear svm. Machine Learn. Res. 11, 1471–1490.

[B4] Cormen T. H. Leiserson C. E. Rivest R. L. Stein C. (2001). Introduction to Algorithms. Cambridge, MA: MIT Press.

[B5] Cui X. Bray S. Bryant D. M. Glover G. H. Reiss A. L. (2010a). A quantitative comparison of nirs and fmri across multiple cognitive tasks. Neuroimage 54, 2808–2821. 10.1016/j.neuroimage.2010.10.069 21047559 PMC3021967

[B6] Cui X. Bray S. Reiss A. (2010b). Functional near infrared spectroscopy (nirs) signal improvement based on negative correlation between oxygenated and deoxygenated hemoglobin dynamics. Neuroimage 49, 30–39. 10.1016/j.neuroimage.2009.11.050 19945536 PMC2818571

[B7] Cui X. Bray S. Reiss A. (2010c). Speeded near infrared spectroscopy (*NIRS*) response detection. PLoS ONE 11:e15474 10.1371/journal.pone.0015474 PMC2978722 21085607

[B8] Dieler A. C. Tupak S. V. Fallgatter A. J. (2012). Functional near-infrared spectroscopy for the assessment of speech related tasks. Brain Lang. 121, 90–109. 10.1016/j.bandl.2011.03.005 21507475

[B9] Fix E. Hodges J. (1951). Discriminatory analysis, nonparametric discrimination: consistency properties. Technical Report_4_, USAF School of Aviation Medicine, Randolph Field, Texas.

[B10] Fletcher P. T. Venkatasubramanian S. Joshi S. (2009). The geometric median on *R*eimannian mainfolds with application to robust atalas estimation. Neuroimage 45, 144–152. 10.1016/j.neuroimage.2008.10.052 19056498 PMC2735114

[B11] Freedman D. (2009). Statistical Models: Theory and Practice. New York, NY: Cambridge University Press.

[B12] Fukunaga K. Koontz W. L. G. (1970). Application of the karhunen-loeve expansion to feature selection and ordering. IEEE Trans. Comput. 19, 311–318. 10.1109/T-C.1970.222918

[B13] Galli F. L. (2014). Powers of tensors and fast matrix multiplication, in Proceedings of the 39th International Symposium on Symbolic and Algebraic Computation (Kobe), 296–303.

[B14] Gazzaniga M. S. Ivry R. B. Mangun G. R. (2014). Cognitive Neuroscience: The Biology of the Mind. New York, NY: W. W. Norton & Company Inc.

[B15] Hai N. Cuong N. Khoa T. Toi V. (2013). Temporal hemodynamic classification of two hands tapping using functional near-infrared spectroscopy. Front. Hum. Neurosci. 7:516. 10.3389/fnhum.2013.00516 24032008 PMC3759001

[B16] Haut K. Barch D. (2006). Sex influences on material-sensetive functional lateralization in working and episodic memory: men and women are not all that different. Neuroimage 32, 411–422. 10.1016/j.neuroimage.2006.01.044 16730459

[B17] Herff C. Heger D. Putze F. Hennrich J. Fortmann O. Schultz T. (2013a). Classification of mental tasks in the prefrontal cortex using f*NIRS*, in Proceedings of IEEE International Conference on Engineering in Medicine and Biology Society (EMBC) (Osaka). 10.1109/EMBC.2013.6609962 24110149

[B18] Herff C. Heger D. Putze F. Hennrich J. Fortmann O. Schultz T. (2013b). Differential entropy feature for *EEG*-based emotion classification, in 6th Annual International IEEE EMBS Conference on Neural Engineering (San Diego, CA), 81–84.

[B19] Herff C. Hegger D. Fortmann O. Hennrich J. Putze F. Schultz T. (2014). Mental workload during n-back task – quantified in the prefrontal cortex using fnirs. Front. Hum. Neurosci. 7:935. 10.3389/fnhum.2013.00935 24474913 PMC3893598

[B20] Holper L. Wolf M. (2011). Single-trial classification of motor imagery differing in task complexity: a functional near-infrared spectroscopy study. J. Neuroeng. Rehabil. 8, 1–13. 10.1186/1743-0003-8-34 21682906 PMC3133548

[B21] Hong K. S. Naseer N. Kim Y. H. (2015). Classification of prefrontal and motor cortex signals for three-class f*NIRS*−*BCI*. Neurosci. Lett. 587, 87–92. 10.1016/j.neulet.2014.12.029 25529197

[B22] Hu X. S. Hong K. S. Ge S. S. (2012). f*NIRS*-based online deception decoding. Neural Eng. 9, 1–12. 10.1088/1741-2560/9/2/026012 22337819

[B23] Kamran M. A. Hong K. S. (2014). Reduction of physiological effects in f*NIRS* waveforms for efficient brain-state decoding. Neurosci. Lett. 580, 130–136. 10.1016/j.neulet.2014.07.058 25111978

[B24] Kang H. Choi S. (2012). Probabilistic models for common spatial patterns: parameter-expanded *EM* and variational bayes, in Proceedings of the Twenty-Sixth AAAI Conference on Artificial Intelligence (Toronto, ON), 970–976.

[B25] Kirchner W. (1958). Age differences in short-term retention of rapidly changing information. J. Exp. Psychol. 55, 352–358. 10.1037/h0043688 13539317

[B26] Kumaran D. Hassabis D. McClelland J. L. (2016a). Investigating critical frequency bands and channels for *EEG*-based emotion recognition with deep neural network. Trends Cogn. Sci. 20, 512–534. 10.1016/j.tics.2016.05.004 27315762

[B27] Kumaran D. Hassabis D. McClelland J. L. (2016b). What learning systems do intelligent agents need? complementary learning systems theory updated. Trends Cogn. Sci. 20, 512–534. 10.1016/j.tics.2016.05.004 27315762

[B28] Li T. Luo Q. Gong H. (2010). Gender-specific hemodynamics in prefrontal cortex during a verbal working memory task by near-infrared spectroscopy. Behav. Brain Res. 209, 148–153. 10.1016/j.bbr.2010.01.033 20117145

[B29] Lin J. (1992). Approximation algorithms for geometric median problems. Inf. Process. Lett. 44, 245–249. 10.1016/0020-0190(92)90208-D

[B30] Lungarella M. Metta G. Pfeifer R. Sandini G. (2003). Developmental robotics: a survey. Connect. Sci. 15, 151–190. 10.1080/09540090310001655110

[B31] Luu S. Chau T. (2009). Decoding subjective preference from single-trial near-infrared spectroscopy signals. Neural Eng. 6, 1–8. 10.1088/1741-2560/6/1/016003 19104138

[B32] Moriai-Izawaa A. Danb H. Dana I. Sanoa T. Ogurob K. Yokotab H.. (2012). Multichannel f*NIRS* assessment of overt and covert confrontation naming. Brain Lang. 121, 185–193. 10.1016/j.bandl.2012.02.001 22429907

[B33] Naito M. Michioka Y. Ozawa K. Ito Y. Kiguchi M. Kanazawa T. (2007). A communication means for totally locked-in *ALS* patients based on changes in cerebral blood volume measured with near-infrared light. IIEICE Tran. Inf. Syst. 7, 1028–1037. 10.1093/ietisy/e90-d.7.1028

[B34] Nakanishi J. Sumioka H. Ishiguro H. (2016). Impact of mediated intimate interaction on education: a huggable communication medium that encourages listening. Front. Psychol. 7:510. 10.3389/fpsyg.2016.00510 27148119 PMC4835693

[B35] Naseer N. Hong K. (2013a). Classification of functional near-infrared spectroscopy signals corresponding to the right- and left-wrist motor imagery for development of a brain-computer interface. Neurosci. Lett. 553, 84–89. 10.1016/j.neulet.2013.08.021 23973334

[B36] Naseer N. Hong K. (2013b). Discrimination of right- and left-wrist motor imagery using f*NIRS*: towards control of a ball-on-a-beam system, in 6th Annual International IEEE EMBS Conference on Neural Engineering (San Diego, CA), 703–706.

[B37] Naseer N. Hong K.-S. (2015a). Decoding answers to four-choice questions using functional near infrared spectroscopy. J. Near Infrared Spectrosc. 23, 23–31. 10.1255/jnirs.1145

[B38] Naseer N. Hong K.-S. (2015b). fNIRS-based brain-computer interfaces: a review. Front. Hum. Neurosci. 9:3. 10.3389/fnhum.2015.00003 25674060 PMC4309034

[B39] Naseer N. Hong M. J. Hong K. S. (2014). Online binary decision decoding using functional near-infrared spectroscopy for the development of brain-computer interface. Exp. Brain Res. 232, 555–564. 10.1007/s00221-013-3764-1 24258529

[B40] Naseer N. Noori F. M. Qureshi N. K. Hong K.-S. (2016). Determining optimal feature-combination of functional near-infrared spectroscopy signals in brain-computer interface application. Front. Hum. Neurosci. 10:237. 10.3389/fnhum.2016.00237 27252637 PMC4879140

[B41] Nguyen T. Ngo Q. Truong Q. Vo V. (2013). Temporal hemodynamic classification of two hands tapping using functional near-infrared spectroscopy. Front. Hum. Neurosci. 7:516. 10.3389/fnhum.2013.00516 24032008 PMC3759001

[B42] Nicholls M. Thomas N. Loetscher T. Grimshaw G. (2013). The flinders handedness survey (*FLANDERS*): a brief measure of skilled hand preference. Cortex 49, 2914–2926. 10.1016/j.cortex.2013.02.002 23498655

[B43] Ogawa K. Nishio S. Koda K. Balistreri G. Watanabe T. Ishiguro H. (2011). Exploring the natural reaction of young and aged person with telenoid in a real world. Int. J. Soc. Robot. 15, 592–597. 10.20965/jaciii2011.p0592

[B44] Power S. Falk T. Chau T. (2010). Classification of prefrontal activity due to mental arithmetic and music imagery using hidden *M*arkov models and frequency domain near-infrared spectroscopy. Neural Eng. 7, 1–8. 10.1088/1741-2560/7/2/026002 20168001

[B45] Prince C. Gogate L. (2007). Epigenetic robotics: behavioral treatments and potential new models for developmental pediatrics. Pediatr. Res. 61, 383–385. 10.1203/pdr.0b013e3180459fdd 17515858

[B46] Shi L. Jiao Y. Lu B. (1995). Random decision forests, in Proceedings of the 3rd International Conference on Document Analysis and Recognition (Montreal, QC), 278–282.

[B47] Shi L. Jiao Y. Lu B. (2013). Differential entropy feature for *EEG*-based vigilance estimation, in IEEE 35th Annual International Conference on Engineering in Medicine and Biology Society (EMBC) (Osaka), 6627–6630. 10.1109/EMBC.2013.6611075 24111262

[B48] Strassen V. (2000). Gaussian elimination is not optimal. Numerische Mathematik 13, 354–356. 10.1007/BF02165411

[B49] Stuart R. Norvig P. (2003). Artificial Intelligence: A Modern Approach. 2nd Edn. Upper Saddle River, NJ: Prentice Hall.

[B50] Sumioka H. Nakae A. Kanai R. Ishiguro H. (2013). Huggable communication medium decreases cortisol levels. Sci. Rep. 3, 1–6. 10.1038/srep03034 24150186 PMC3805974

[B51] Sweller J. (1988). Cognitive load during problem solving: effects on learning. Cogn. Sci. 12, 257–285. 10.1207/s15516709cog1202_4

[B52] Tai K. Chau T. (2009). Single-trial classification of nirs signals during emotional induction tasks: towards a corporeal machine interface. J. Neuroeng. Rehabil. 6, 1–14. 10.1186/1743-0003-6-39 19900285 PMC2779792

[B53] Tanaka F. Cicourel A. Movellan J. (2007). Socialization between toddlers and robots at an early childhood education center. Proc. Natl. Acad. Sci. U.S.A. 104, 17954–17958. 10.1073/pnas.0707769104 17984068 PMC2084278

[B54] Weiss E. Siedentopf C. Hofer A. Deisenhammer E. Hoptman M. Kremser C. (2003). Sex influences on material-sensetive functional lateralization in working and episodic memory: men and women are not all that different. Neurosci. Lett. 344, 169–172. 10.1016/S0304-3940(03)00406-3 12812832

[B55] Xu B. Fu Y. Shi G. Yin X. Wang Z. Li H. (2014). Improving classification by feature discretization and optimization for f*NIRS*-based *BCI*. Biomimet. Biomater. Tissue Eng. 19, 1–5. 10.4172/1662-100X.1000119

[B56] Yamazaki R. Christensen L. Skov K. Chang C. Damholdt M. Sumioka H.. (2016). Intimacy in phone conversations: anxiety reduction for danish seniors with hugvie. Front. Psychol. 7:537. 10.3389/fpsyg.2016.00537 27148144 PMC4835483

[B57] Yamazaki R. Nishio S. Ishiguro H. Nazrskov M. Ishiguro N. Balistreri G. (2014). Acceptability of a teleoperated android by senior citizens in danish society: a case study on the application of an embodied communication medium to home care. Int. J. Soc. Robot. 6, 429–442. 10.1007/s12369-014-0247-x

[B58] Yamazaki R. Nishio S. Ogawa K. Matsumura K. Minato T. Ishiguro H. (2007). Promoting socialization of school children using a teleoperated android: an interaction study. Int. J. Hum. Robot. 10, 1350007(1–25) 10.1142/S0219843613500072

