# Supplementary material for: A Non-parametric Approach to the Overall Estimate of Cognitive Load Using NIRS Time Series
Source: Front Hum Neurosci. 2017 Feb 3;11:15. doi: 10.3389/fnhum.2017.00015 (PMC5290219; doi:10.3389/fnhum.2017.00015)
Supplement: Supplementary file 1 [file DataSheet1.pdf]

SUPPLEMENTARY MATERIALS
Article Entitled ”A Nonparametric Approach to the Overall Estimate of Cognitive Load Using NIRS Time Series”

Soheil Keshmiri [1,*], Hidenobu Sumioka [1], Ryuji Yamazaki [1] and Hiroshi Ishiguro [1,2]

[1] *Hiroshi Ishiguro Laboratories (HIL), Advanced Telecommunications Research Institute International (ATR), Kyoto, Japan.*
[2] *the Graduate School of Engineering Science, Osaka University*

Correspondence*:
Soheil Keshmiri
soheil@atr.jp

1 INTRODUCTION

2 METHDOLOGY

3 COMPUTATION OF THE DIFFERENTIAL ENTROPY (DE) FEATURE

DE generalizes the concept of entropy for discrete random variables to the realm of continuous random variable. DE of a continuous random variable X is calculated as (Kumaran et al., 2016):

$$h(X) = -\int_X f(x)log(f(x))dx \tag{1}$$

Furthermore, when $X \sim \mathcal{N}(\mu, \sigma^2)$, equation (1) simplifies into:

$$h(X) = -\int_{-\infty}^{\infty} \frac{1}{\sqrt{2\pi\sigma^2}} e^{\frac{(x-\mu)^2}{2\sigma^2}} log(\frac{1}{\sqrt{2\pi\sigma^2}} e^{\frac{(x-\mu)^2}{2\sigma^2}})\partial x = \frac{1}{2}log(2\pi e\sigma^2) \tag{2}$$

It is worth noting that the desired behaviour to follow the normal distribution is obtained via normalization of the NIRS data, thereby acquiring its standard normal distribution $X \sim \mathcal{N}(0, 1)$.

4 SUPPLEMENTARY DEFINITIONS AND CLAIMS

Definition 4.1. Given N points, $p_1, \ \ldots, \ p_N \in \mathbb{R}^n \ (n \geq 1)$, there exists a point $x \in \mathbb{R}^n \ (n \geq 1)$ that minimizes the function (Boltyanski et al., 1999):

$$f(x) = \sum_{i=1}^{N} w_i \|\vec{p}_i - x\|, \ \vec{p}_1, \ldots, \vec{p}_N \in \mathbb{R}^n \ (n \geq 1) \tag{3}$$

with $w_i,\ i = 1, \ldots, N$ being the weight associated with i^{th} data point and x is the geometric median (a.k.a generalized Fermat-Torricelli point or the Weber point) of $p_1, \ldots, p_N$ (Boltyanski et al., 1999).

CLAIM 4.1. *The function f is convex (Boltyanski et al., 1999).*

CLAIM 4.2. *The function f is strictly convex function if and only if the points $p_1,\ \ldots,\ p_N \in \mathbb{R}^n$ $(n \geq 1)$ are not collinear (Boltyanski et al., 1999).*

5 OVERALL COMPLEXITY OF THE PROPOSED APPROACH

It is apparent that equations (1) and (2) are linear in the size of the input data. Furthermore, equations (4) and (5) are $O(1)$ procedures, followed by the equation (6) that is $O(M)$, with M representing the size of the test set. Moreover, the latter becomes $O(1)$ in case of realtime prediction where the system requires to estimate the state of an individual based on the NIRS time series associated with the prediction interval. On the other hand, equation (3) introduces higher degree of complexity due to the matrix-matrix multiplication in its formulation. More specifically, the first term in this equation requires the inverse of the input feature matrix that is multiplied by its transpose, indicating an $O(N^3)$ computation with N being the size of the input. Whereas, the second term stays linear in the size of input i.e., $O(N)$ since it involves a matrix-vector multiplication.
However, there are three observations to make:

1. Equation (3) reduces the overall computation of the weights associated with our model into one step, resulting in an overall $O(1)$ complexity that is independent of the size of the input or the nature of the data involved. This is a highly desirable feature for a learning model once deployed in a real world setting. (Surname, 2002)
2. The aforementioned $O(1)$ overall complexity becomes more attractive considering the fact that feature spaces associated with NIRS data are commonly low dimensional.
3. It is possible to speed up the computation of the first term in equation (3) via application of faster matrix multiplication technqiues such as Strassen algorithm (Strassen, 2000), achieving an $O(N^{2.81})$. Furthermore, the recent modification of this algorithm scales down its complexity to $O(N^{2.372})$ (Galli, 2014).

REFERENCES

Boltyanski, V., Martini, H., and Soltan, V. (1999). *Geometric Methods and Optimization Problems* (Kluwer Academic, Boston)

Galli, F. L. (2014). Powers of tensors and fast matrix multiplication. In *Proceedings of the 39th International Symposium on Symbolic and Algebraic Computation*. 296–303

Kumaran, D., Hassabis, D., and McClelland, J. L. (2016). Investigating critical frequency bands and channels for EEG-based emotion recognition with deep neural network. *Trends in Cognitive Science* 20, 512–534

Strassen, V. (2000). Gaussian elimination is not optimal. *Numerische Mathematik* 13, 354–356

Surname, B. (2002). The title of the work. In *The title of the book*, ed. E. Name (The city: The name of the publisher). 201–213
